# Does atrial fibrillation increase the risk of fractures? A systematic review and meta-analysis

**DOI:** 10.3389/fmed.2025.1528195

**Published:** 2025-05-27

**Authors:** Wei Chen, Yuyu Zhang, Huaze Xie, Haiyi Guo, Yangfan Gong, Zhuohao Yin, Kai Zhao, Wei Ge

**Affiliations:** ^1^Department of General Practice, Xijing Hospital, The Fourth Military Medical University, Xi’an, Shannxi, China; ^2^Department of Cardiology, Xijing Hospital, The Fourth Military Medical University, Xi’an, Shannxi, China; ^3^Department of Medical Information, Ningxia Medical University, Yinchuan, Ningxia, China; ^4^Sargent College of Health and Rehabilitation Science, Boston University, Boston, MA, United States

**Keywords:** atrial fibrillation, fracture, cohort study, meta-analysis, Asians

## Abstract

**Objectives:**

We have observed in clinical practice that patients with fractures often have concomitant atrial fibrillation. However, it remains unclear whether atrial fibrillation increases the risk of bone fracture. A meta-analysis was performed to investigate the association between atrial fibrillation and fracture.

**Methods:**

PubMed and Cochrane Library were searched for relevant studies from 1 January 1943 to 31 December 2024 that compared the prevalence of fracture in atrial fibrillation group with non-atrial fibrillation group.

**Results:**

A total of five cohort studies with 187,868 participants met all the eligibility criteria for our study. A total of 835 people suffered a fracture in atrial fibrillation group and 6,512 in non-atrial fibrillation group. The overall risk of fractures was non-statistically higher in patients with 5.4% (835/15,395) in atrial fibrillation group and 3.8% (6,512/172,473) in non-atrial fibrillation group. Analysis of included studies observed non-significant association between atrial fibrillation and fractures [odds ratio (OR) = 1.17, 95% confidence interval (CI) = 0.60–2.29, *P* = 0.65]. However, subgroup analysis displayed that Asian population with atrial fibrillation had a higher risk of fracture (OR = 1.61, 95% CI = 1.38–1.87, *P* < 0.00001), whereas no similar outcomes were seen in Caucasian population (OR = 0.94, 95% CI = 0.24–3.59, *P* = 0.92).

**Conclusion:**

The evidence indicated that Asians with atrial fibrillation were more prone to fractures.

**Systematic review registration:**

https://www.crd.york.ac.uk/PROSPERO/myprospero, identifier CRD42018107794.

## 1 Introduction

With the increase of the global older adult population, fractures have become increasingly prevalent worldwide. Approximately 8.9 million low energy fractures occur annually around the globe (1). Fractures cause significant disability, compromise quality of life, increase medical costs and mortality. Given the increasing economic burden, incidence rates, and mortality associated with fractures, identifying modifiable risk factors that contribute to fracture risk becomes crucial for developing effective prevention strategies and mitigating the public health impact of osteoporotic fractures.

Emerging evidence suggests a potential association between cardiovascular diseases and elevated fracture risk, with established data demonstrating increased fracture incidence in patients with heart failure and myocardial infarction (2). AF is one of the most common arrhythmias in the older adult (3, 4), studies have shown that AF can increase the risk of fractures (5, 6). Pathophysiological mechanisms may involve AF-induced hypotension, dizziness, and reduced cerebral perfusion that heighten fall susceptibility. Furthermore, AF patients frequently experience cerebral infarction and accelerated cognitive decline, both linked to impaired gait mechanics, recurrent falls, and hip fracture risk. Pharmacological considerations add complexity, as long-term warfarin therapy in AF patients – while preventing thromboembolism – may reduce bone mineral density through vitamin K antagonism. However, observational studies have yielded conflicting findings, with some reporting no significant fracture risk elevation in AF populations (7, 8). This contradictory evidence underscores the necessity for prospective cohort studies and mechanistic investigations to elucidate the causal pathways and clinical implications of AF-fracture relationships, particularly regarding fall-related fracture prevention strategies in aging populations.

This systematic review and meta-analysis aim to compile the findings of available literature and determine the cumulative association of AF as a risk factor for non-traumatic, non-accidental fractures.

## 2 Methods

This systemic review and meta-analysis were conducted following the guidelines of Meta-analysis of Observational Studies in Epidemiology (MOOSE) (9) and the Preferred Reporting Items for Systematic Review and Meta-Analysis (PRISMA) (10). The study was registered in PROSPERO, where a detailed protocol can be obtained, and the registration number is CRD42018107794.

### 2.1 Searching strategy

PubMed and Cochrane Library were searched for eligible literatures from 1 January 1943 to 31 December 2024. A researcher trained by a qualified librarian created the searching strategy for PubMed and Cochrane Library. Searching strategies for mentioned databases were shown in the appendix. References of included studies were also reviewed.

### 2.2 Inclusion criteria and exclusion criteria

Based on the PICO-SD framework, our study population comprised adult patients (≥ 18 years) with a confirmed atrial fibrillation (AF) diagnosis, irrespective of AF subtype or CHA2DS2-VASc score. AF was defined as the exposure variable, while age- and sex-matched individuals without AF served as controls. The primary outcome was composite fracture risk (any fracture site). Eligible study designs included prospective and retrospective cohort studies, nested case-control studies, and randomized controlled trials (RCTs) reporting fracture outcomes in AF populations. A systematic search was conducted across PubMed and Cochrane Library databases to identify relevant literature. Only studies published in English were included in the analysis. Articles that did not discuss or report numbers of patients with AF were excluded. Additionally, conference papers were excluded due to inconclusive results. Any fractures that caused by trauma, height falls and motor vehicle accident were excluded.

### 2.3 Data extraction and quality assessment

Two authors independently reviewed titles and abstracts to assess studies for inclusion. Three reviewers independently abstracted data using a standardized datasheet. The information on following variables were abstracted: author or investigator name, year of publication, race, location, number of participants with and without AF, number of participants with incident fractures with or without AF, mean duration of follow-up, study design, mean age of participants with and without AF, fracture site. Study quality was evaluated by Newcastle-Ottawa Scale (NOS), which was consisted of three aspects containing selection, comparability and outcome (11). Studies with Scores more than six was considered as high quality in our review. Any Discrepancies between reviewers for inclusion of studies and data extraction were discussed, regarding the inclusion of studies and data extraction, and a consensus decision was reached (Supplementary Tables 1–3).

### 2.4 Statistical analysis

RevMan 5.4 software, developed by the Cochrane Collaboration (accessed on 20th June 2021), was used for this systematic review and meta-analysis. The pooled meta-analysis of the cohort studies was performed using the Mantel-Haenszel method to calculate the odds ratio (OR) and 95% confidence interval (CI). Heterogeneity in the total variation of effect across studies was assessed using Higgins I^2^ statistics. When *P* > 0.1 and I^2^ < 50%, no heterogeneity was considered present, whereas when *P* < 0.1 and I^2^ > 50%, heterogeneity was considered significant. A fixed-effect model was used for I^2^ below 50%, and a random-effects model was used for I^2^ above 50% (12). Additionally, subgroup analysis of studies, such as race, was performed to explore the source of heterogeneity in greater detail. Sensitivity analysis was also conducted by removing one study at a time to observe any changes in the odds ratio, *p*-value, and the overall outcome, aiming to identify the disproportionate influence of any specific study on the results (Supplementary Tables 1–3).

### 2.5 Patient involvement

Patients were not involved at any stage of the study, including the definition of the research question, selection of outcome measures, study design, or implementation of the study. Furthermore, patients did not contribute to the interpretation or writing up of the study. The research was conducted without direct patient involvement.

## 3 Results

### 3.1 Literature search and characteristics of included studies

**FIGURE 1:**
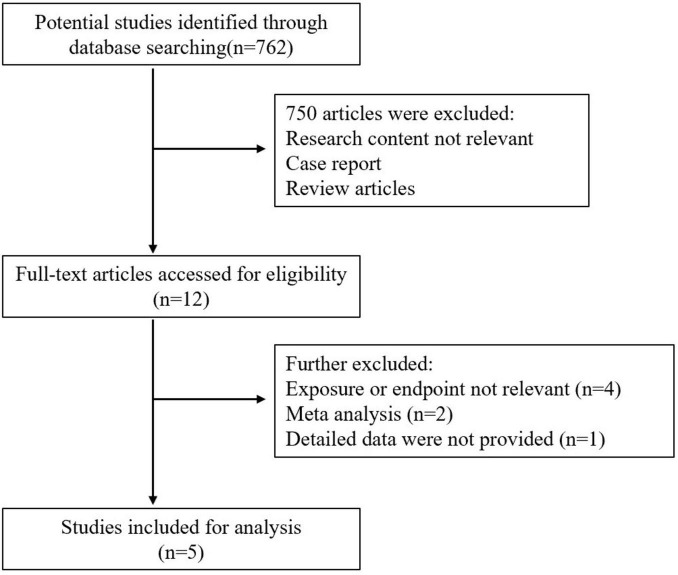
Flow diagram of search strategy and selection of studies.

A Total of 762 studies were yielded from databases searches. All studies were screened by title and abstract. Among them, 750 studies were excluded, and 12 studies were retrieved for a full text review. Finally, only five studies met the eligibility and were analyzed in the systematic review and meta-analysis. These five articles are retrospective studies reporting the incidence of fractures in patients with atrial fibrillation. The Flow diagram of the search strategy was depicted in Figure 1. Detailed general characteristics of all included studies were provided in Table 1.

**TABLE 1:** Baseline characteristic of included studies.

Study author	Year of publication	Design of study	Location of study	Race	Mean age (years)	Female (%)	Anticoagulant (warfarin %)	BMI	Numbers of participants	Numbers of incidence of fractures in AF	Numbers of incidence of fractures in non-AF	Mean follow-up period (years)	Site of fracture
Hui-Chin Lai	2015	Cohort Study	Taiwan	Asian	74	42.2	29.1	–	34,625	163 (6,925)	379 (27,700)	3.6	Skull fracture, neck and trunk fractures, and lower limb fracture
Christopher X. Wong	2017	Cohort Study	Australia	Caucasian	55	51.5	–	26.6	113,600	361 (5563)	2,560 (108,037)	14	Hip fracture
Erin R. Wallace	2017	Cohort Study	United States	Caucasian	74	45.6	–	–	4,462	97 (1,007)	620 (3,455)	8.8	Hip fracture, distil forearm fracture, humerus fracture and pelvis fracture
Daehoon kim	2018	Cohort Study	Korea	Asian	69	85	23.7	24.4	31,778	142 (1,213)	2,500 (30,565)	4	Hip fracture, vertebral fracture, pelvic fracture, and acetabular fracture
Jason A. Sherer	2020	Cohort Study	United States	Caucasian	68	53.2	–	27.5	3,403	72 (687)	453 (2,716)	12.5	All fractures excluding fractures of the finger, toe, foot, skull, and facial bones.

### 3.2. Synthesis of study results

**FIGURE 2:**
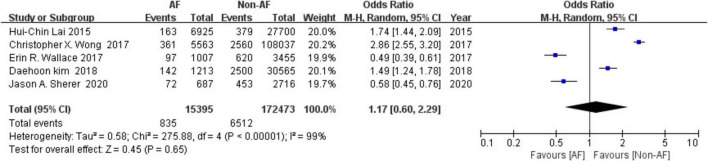
Forest plot of association between atrial fibrillation with fracture.

There were total of 187,868 patients in five included studies including 15,395 in the AF group and 172,473 in the non-AF group. Among them, 835 people suffered a fracture in the AF group, while 6,512 people experienced fractures in the non-AF group. Statistically, the overall risk of fractures was non-significantly higher in patients with AF at 5.4% (835/15,395) compared to 3.8% (6,512/172,473) in the non-AF group (OR = 1.17, 95% CI = 0.60–2.29 *P* = 0.65) (Figure 2).

### 3.3 Association of fracture with atrial fibrillation in different population

**FIGURE 3:**
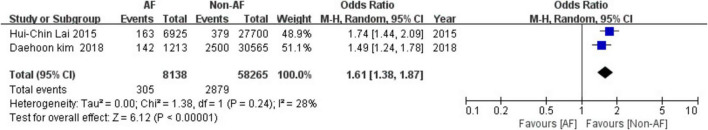
Forest plot of association between atrial fibrillation with fracture in Asian population.

Based on demographic characteristics, patients were divided into Asian and Caucasian populations, and the statistical results indicated that Asian population had a significant higher risk of fractures in population in the AF group compared with non-AF group (OR = 1.61, 95% CI = 1.38–1.87, *P* < 0.00001) and no statistically heterogeneity (I^2^ = 28%, *P* = 0.24) was observed after merging Asian studies (Figure 3).

**FIGURE 4:**
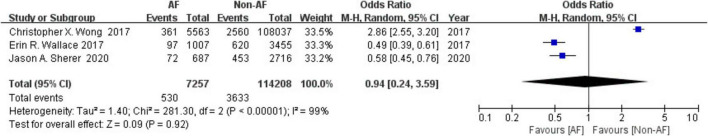
Forest plot of association between atrial fibrillation with fracture in Caucasian population.

The association of low energy fractures with AF in the Caucasian population remains inconclusive (OR = 0.94, 95% CI = 0.24–3.59, *P* = 0.92). High heterogeneity was observed (*P* < 0.00001) after combining the included Caucasian studies. However, when the study by Christopher X. Wong was excluded, the heterogeneity decreased significantly (I^2^ = 5%, *P* = 0.3) (Figure 4).

## 4 Discussion

There was a complex relationship between AF and fracture risk in aging populations. While both conditions demonstrate significant prevalence in geriatric patients, our comprehensive analysis failed to establish a direct causal relationship between AF and increased fracture susceptibility in the general population. Notably, subgroup analysis uncovered significant demographic disparities: Asian cohorts exhibited a elevation in fracture risk among AF patients (OR = 1.61, 95% CI = 1.38–1.87, *P* < 0.00001).

### 4.1 Studies on the risk of low energy fractures in patients with AF

Wallace et al. (7), Sherer et al. (8) had reported that individuals with AF were not at higher risk of fractures. In contrast, other three studies revealed that more frequency of fractures in people with AF than non-AF. The opposite conclusions drawn from different studies may be related to the following reasons. Firstly, the number of participates ranges from 3,403 to 113,600 and three studies were on Caucasian population and two on Asian. Secondly, median follow-up period was 14 years in Jason A. Sherer study and 12.5 years in Wong et al. (25) study while studies by Hui Chin Lai, Daehoon kim, and Erin R. Wallace were 3.6, 4, and 8.8 years, respectively. In consideration of study designs and different follow-up times, recall bias and selection bias could not be avoided in this study. Furthermore, site of fracture differed in five studies. A previous study by Rabieh Abu-Assi evaluated the association of AF and hip fracture, which indicated an unconclusive results about whether patients with AF was related to raise risk of hip fracture (13). What is more, differences such as medical history of heart failure, stroke and anticoagulant drugs use in baseline characteristics also conduced to the obvious heterogeneity in our review. The medical history of heart failure and stroke was related with increased subsequent fracture. Individuals of heart failure and stroke history were excluded by the study of Wallace et al. (7) and not mentioned in study published in 2020, while those history were contained in other three included studies. The study of Sherer et al. (8) reported a significant correlation of low energy fractures with AF (HR = 1.37, 95% CI = 1.06–1.79) at the index age of 65 years. However, baseline characteristic of this study included the participates with fractures history and after adjusting this covariate, AF was no longer significantly associated with fracture during follow-up period. As to anticoagulant drugs use, only two studies take it into consideration and results were opposite. Previous studies have suggested these covariates might decrease bone mineral density and raise the risk of fractures (14–16). Finally, all included studies had limitations which could have contributed to the confounding of their outcome (Supplementary Table 4).

### 4.2 The possible rationale behind the association of fracture with AF

**FIGURE 5:**
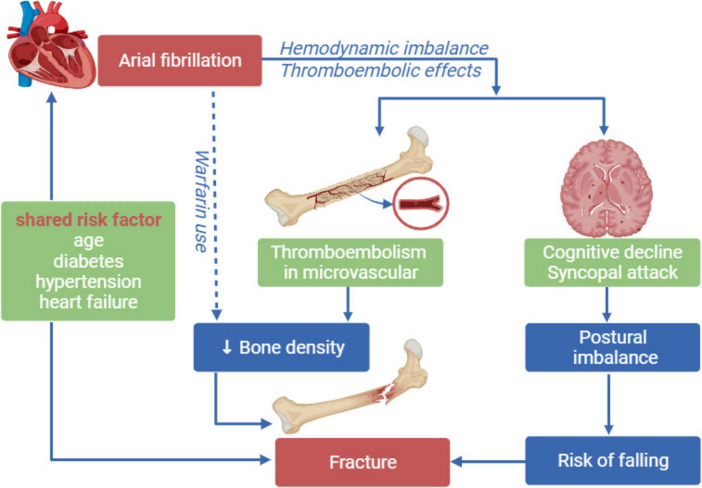
The possible relationship between atrial fibrillation and fractures.

Chronic atrial fibrillation causes decreased cardiac output, fluctuation of blood pressure and declined organ perfusion and multiorgan thromboembolism (6, 17). Blood supply of central nervous system is disordered by hemodynamic imbalance and thromboembolism, which leads to abnormal cerebral perfusion, cognitive decline and increased syncopal attacks, and further causes postural imbalance and increasing the tendency to fall (18). Besides, accelerated decrease in cognition, higher brain infarction, and white matter abnormalities have been frequently seen in patients with AF (19, 20). All these brain lesions impair gait and increase risk of fall (21). Additionally, bone mineral density is also decreased due to the impaired blood supply to bone and thromboembolism of bone microvascular, which makes the bone susceptible to fracture (5). Furthermore, warfarin is frequently provided to patients with AF to prevent thromboembolism. Although warfarin is well-studied to decrease bone mineral density by inhibiting carboxylation of osteocalcin which is an important factor in maintenance of bone mineral density (22, 23), risk of lower bone density of long-time used warfarin in AF individuals is still conflicted (24). In addition, risk factors such as age, diabetes, hypertension, and heart failure were both related to AF and fracture (25). As to diabetics, bone quality is decreased for chronic hyperglycemia by inhibiting osteocalcin, increasing reactive oxygen species, accumulating advanced glycation end products in bone, or inhibiting IGF-1. Also, diabetic complications can increase risk of falls (26). As to heart failure population, increased excretion of calcium for secondary hyperparathyroidism and lower vitamin D levels are linked with low bone density (8). To summarize, the possible mechanisms linking AF and subsequent fracture involve two major factors: falling incidents and decreased bone mineral density (Figure 5).

### 4.3 Possible cause of significant association of AF in Asian studies

The results from the Asian population in our systematic review suggest a positive outcome, indicating that AF might indeed be a risk factor for fractures in this ethnic group. The association between fractures and AF in the Asian population could potentially be attributed to the effects of warfarin usage and metabolic genes. Warfarin was commonly prescribed to patients with AF to reduce the risk of thromboembolism (27, 28). Mount of studies have shown that long term use of warfarin increases the risk of fracture. Several animal studies also discovered that warfarin decreased bone mineral density and increased trabecular separation in femur and vertebrae (29, 30). The difference in the metabolism of warfarin has been found in different races (31). This difference in metabolism of warfarin was mainly determined by genetic polymorphism of CYP2C9 (Cytochrome P450 2C9) and VKORC1 (Vitamin K epoxide reductase complex subunit 1). Asian population has been found to carry more CYP2CP*1 and VKORC1 GC gene type than Caucasians, which leads to decrease the metabolism of warfarin and greater plasma levels of warfarin for a long time in the Asian population, resulting in decreased bone mineral density and a higher risk of fractures (32, 33). However, Asian studies included in our systematic review did not provide adjusted data of fractures in patients with AF who were compliant with warfarin. In order to rule out the confounding effect of warfarin on significant association of low energy fractures with AF, an adjusted hazard ratio of fractures with respect to warfarin utilization is needed.

### 4.4 Limitations

Some of the limitations of our systematic review were as follows: (1). All included cohort studies recruited participants from databases which were subjected to systemic and random errors and inconsistencies. (2). Majority studies were retrospective, which did not address several risk factors that were associated with fractures. Prospective cohort studies have not been done. There is a need to study the association of AF and low energy fracture in patients who are not on anticoagulant or non-complaint with AF. (3). Studies are lacking in some races, for example, African population was not involved in any of the study. So results are not generalizable. (4). Included studies were not adjusted for all possible risk factors of fractures. (5). Only studies published in English were included in the review which raised chances of missing some of the studies. (6). The substantial heterogeneity could not be explored completely due to the unavailability of several important factors such as vitamin D levels. (7). Publication bias and the lacking of availability of individual data were existed in any meta-analysis.

Clinical importance and direction for further research.

In primary health care, we should screen the risk factors of fractures in the older adult Asian population with atrial fibrillation, carry out fall related fracture education and strengthen preventive measures in the community, so as to avoid low-energy fractures in patients with atrial fibrillation. These measures are beneficial to reduce the incidence rate and medical expenses of fractures and improve the quality of life of the older adult. It is an important task for primary health care providers. More studies need to be conducted which address bone mineral density, vitamin D and calcium supplements and physical activity levels of individuals. Study designs should also include the duration of warfarin use in AF because of bone mineral density decrease with the increase in the duration of warfarin use. Besides, designs on Caucasian, African, and other races were essential.

## 5 Conclusion

Based on the available literature, our systematic review and meta-analysis concludes that AF is not associated with fracture in all ethnicities combined. However, a significant association of AF with fractures was observed in the Asian population. Results of this meta-analysis remain inconclusive and demand more studies to reach consensus on the link of AF with fractures. In order to acquire conclusive results, prospective cohort studies should be conducted around the globe on multiple ethnicities to evaluate interrelation of AF on fractures.

## Data Availability

The raw data supporting the conclusions of this article will be made available by the authors, without undue reservation.
