# Supplementary material for: Does atrial fibrillation increase the risk of fractures? A systematic review and meta-analysis
Source: Front Med (Lausanne). 2025 May 27;12:1528195. doi: 10.3389/fmed.2025.1528195 (PMC12149180; doi:10.3389/fmed.2025.1528195)
Supplement: Supplementary file 1 [file Table_1.docx]

| **Supplementary table 1: Quality scores of included studies using Newcastle-Ottawa Scale** | | | | | | | | | | |
| --- | --- | --- | --- | --- | --- | --- | --- | --- | --- | --- |
| **study** | **Selection** | | | | | **Comparability** | | **outcome** | | **NOS** |
|  | **representativeness of the expose cohort** | **Selection of the non- exposed cohort** | **Ascertainment of atrial fibrillation** | | **Demonstration that outcomes was not present at start of study** | **Comparability on the basis of the design or analysis** | **Assessment of outcome** | **Adequate follow-up duration** | **Adequate follow-up rate** | **overall scores** |
| Hui-Chin Lai | 1 | 1 | | 1 | 1 | 2 | 1 | 1 | 1 | 9 |
| Christopher X. Wong | 1 | 1 | | 1 | 1 | 1 | 1 | 1 | 1 | 8 |
| Erin R. Wallace | 1 | 1 | | 1 | 0 | 2 | 1 | 1 | 1 | 8 |
| Daehoon kim | 1 | 1 | | 1 | 1 | 2 | 1 | 1 | 1 | 9 |
| Jason A. Sherer | 1 | 1 | | 1 | 0 | 2 | 1 | 1 | 1 | 8 |

| **Supplementary table 2: Sensitivity analysis for all included studies** | | | | |
| --- | --- | --- | --- | --- |
| **Excluding study** | **OR and 95% CI** | **P-value** | **Heterogeneity（%）** | **P-value for heterogeneity** |
| Hui-Chin Lai | 1.05 (0.44, 2.55) | 0.91 | 99% | P<0.00001 |
| Christopher X. Wong | 0.93 (0.50, 1.74) | 0.82 | 97 | P<0.00001 |
| Erin R. Wallace | 1.45 (0.80, 2.63) | 0.22 | 98 | P<0.00001 |
| Daehoon kim | 1.10 (0.45, 2.67) | 0.84 | 99 | P<0.00001 |
| Jason A. Sherer | 1.38 (0.69, 2.79) | 0.36 | 99 | P<0.00001 |

| **Supplementary table 3: Sensitivity analysis for all Caucasian population** | | | | |
| --- | --- | --- | --- | --- |
| **Excluding study** | **OR and 95% CI** | **P-value** | **Heterogeneity（%）** | **P-value for heterogeneity** |
| Christopher X. Wong | 0.53 (0.44, 0.63) | P<0.00001 | 5 | 0.3 |
| Erin R. Wallace | 1.30 (0.26, 6.47) | 0.75 | 99 | P<0.00001 |
| Jason A. Sherer | 1.18 (0.19, 7.21) | 0.85 | 100 | P<0.00001 |

**Supplementary table 4: Limitations of included studies**

| **Study Author** | **Limitations** |
| --- | --- |
| Hui-Chin Lai | Minor bone fracture without the need for hospitalization were not collected in National Health Insurance Research Database (NHIRD) of Taiwan; All cases of osteoporosis were not documented in National Health Insurance Research Database (NHIRD) of Taiwan; Data on time in therapeutic range was not included in NHIRD so proportion of days covered for anticoagulant were included to represent treatment adequacy of oral anticoagulant; Causal link could not be established between fracture and osteoporotic fractures. |
| Christopher X. Wong | Study was not adjusted for all possible risk factors of osteoporotic fracture e.g. body weight, bone mineral density, and vitamin D levels; Delay in diagnosis of atrial fibrillation due to its episodic nature; Use of databases which is subjected to errors and inconsistencies; Underestimation of medications and comorbidities because dataset could not linked to primary care source and hospital data; Results were not generalizable due to majority of participants were Caucasians. |
| Erin R. Wallace | Under ascertainment of incident atrial fibrillation due to its episodic nature; Use of administrative data with random and systemic errors; Included cases of atrial fibrillation were not categorized into persistent or paroxysmal; Atrial fibrillation was not categorized into paroxysmal or persistent. |
| Daehoon kim | Study was not adjusted for all possible risk factors of osteoporotic fracture e.g. bone mineral density, vitamin D levels, falls, frailty , and non-vitamin K antagonist oral anticoagulant prescription; Administrative dataset subjected to errors and inaccuracies; Included cases of atrial fibrillation were not categorized into persistent or paroxysmal; Minor bone fractures not requiring hospitalization were not included; No causality was established due to observational nature of study; Only Asian population was included in the study. |
| Jason A. Sherer | There may be misclassification between the atrial fibrillation and the non-atrial fibrillation; The ascertainment of non-hip fractures prior to 2002 was not confirmed via medical records; Included cases of atrial fibrillation were not categorized into persistent or paroxysmal or others; The participants of the study were mostly European ancestry. |
